# Effect of CYP2C19*17 gene polymorphism on plasma levels of diazepam and nordiazepam in Turkish patients with Alcohol Withdrawal Syndrome

**DOI:** 10.1192/j.eurpsy.2025.637

**Published:** 2025-08-26

**Authors:** S. Özkan-Kotiloğlu, D. Kaya-Akyüzlü, M. A. Yıldırım, M. Danışman, C. Bozmaoğlu, K. C. Tok, M. Gümüştaş, I. Özgür-İlhan, S. Süzen

**Affiliations:** 1 Molecular Biology and Genetics, Kırşehir Ahi Evran University, Kırşehir; 2 Institute of Forensic Sciences, Ankara University; 3 AMATEM Clinic, Ankara Training and Research Hospital; 4 Department of Mental Health and Diseases; 5 Department of Pharmaceutical Toxicology, Ankara University, Ankara, Türkiye

## Abstract

**Introduction:**

Alcohol withdrawal syndrome (AWS) is not a common medical condition in general population however it affects patients with alcohol use disorder (AUD) and causes severe complications when diagnosed late or left untreated. Diazepam is a benzodiazepine, which is used to treat various diseases such as insomnia, anxiety, muscle spasm, pain and AWS. Compared to other benzodiazepines, diazepam is more efficient to prevent delirium and decrease withdrawal due to its long half-life. Diazepam is metabolised to its main metabolite nordiazepam with the enzymes expressed by *CYP2C19* and *CYP3A4* genes. It has been reported that metabolic activity of the enzymes encoded by *CYP2C19* gene may be varied due to genetic polymorphisms leading a change in the efficiency of treatment via effecting the plasma level of drugs metabolised by *CYP2C19.*

**Objectives:**

The aim of this study is to investigate whether CYP2C19*17 gene polymorphism has an impact on plasma levels of diazepam and nordiazepam in the Turkish patients with AWS and under oral diazepam treatment.

**Methods:**

The study included 50 male patients who were in withdrawal state and taking diazepam therapy. CYP2C19*17 polymorphism was determined by PCR-RFLP method. Plasma levels of diazepam (DZP) and nordiazepam (NDZP) were detected by HPLC.

**Results:**

Genotype frequencies were calculated as 66% for CC, 30% for CT and 4% for TT. Dose-normalized DZP and dose-normalized NDZP values were 0.049 μg/ml per mg/day and 0,056 μg/ml per mg/day, respectively. No statistical significance was observed in the levels of normalized DZP and NDZP when CC and CT+TT genotypes were compared (*p*= 0.073 and *p*=0.282, respectively).

**Conclusions:**

The effect of CYP2C19*17 polymorphism on the plasma levels of DZP and NDZP following long term oral diazepam to treat patients with AWS was determined for the first time. With the help of current study, first data on Turkish population was obtained and may be useful for personalized therapy in the future.

*This study was supported by Scientific and Technological Research Council of Turkey (TUBITAK) under the Grant Number 121C441. The authors thank to TUBITAK for their supports.*

**Disclosure of Interest:**

None Declared

